# Insecticide-treated net utilization and associated factors among pregnant women in Ethiopia: a systematic review and meta-analysis

**DOI:** 10.3389/fgwh.2023.1147583

**Published:** 2023-11-06

**Authors:** Bajrond Eshetu, Habtamu Bekele, Adera Debella, Addis Eyeberu, Bikila Balis, Sisay Habte, Kibiru Mardasa, Fenta Wondimneh, Tilahun Teshager, Indeshaw Ketema

**Affiliations:** ^1^ Department of Midwifery, School of Nursing and Midwifery, College of Health and Medical Sciences, Haramaya University, Harar, Ethiopia; ^2^ Department of Nursing, School of Nursing and Midwifery, College of Health and Medical Sciences, Haramaya University, Harar, Ethiopia; ^3^ Adama Hospital Medical College, Adama, Ethiopia; ^4^ Department of Emergency and Critical Care Nursing, School of Nursing and Midwifery, College of Health and Medical Sciences, Haramaya University, Harar, Ethiopia

**Keywords:** insecticide-treated net, pregnant women, systematic review, meta-analysis, Ethiopia

## Abstract

**Background:**

Malaria is a major public health problem in many developing countries, particularly in sub-Saharan Africa. The pregnant woman, fetus, and newborn infant are all at risk from malaria during pregnancy. Hence, insecticide-treated bed net (ITN) use is the most effective and advisable method for preventing malaria during pregnancy. Studies on the prevalence of ITN utilization among pregnant women in Ethiopia are inconsistently reported and highly varied. Therefore, this systematic review and meta-analysis aimed to estimate the pooled prevalence of ITN utilization and associated factors among pregnant women in Ethiopia.

**Methods:**

A comprehensive search of databases such as PubMed, CINAHL, Web of Science, SCOPUS, Science Direct, Google, and Google Scholar was performed to find studies conducted in Ethiopia. All original observational studies that reported the prevalence of ITN utilization were identified and screened. The Newcastle-Ottawa scale tool was used to assess the quality of the studies. Data were extracted in Microsoft Excel 2010 format and analyzed using STATA Version 14. A random-effect meta-analysis model was utilized to estimate the pooled prevalence of ITN utilization. The statistical heterogeneity was checked using the *I*^2^ test and subgroup analysis. The publication bias was assessed using funnel plots and Egger's regression test. The size of the pooled effect of the factors influencing the use of ITNs was estimated using an odds ratio (OR) with a 95% confidence interval (CI), and a *P*-value <0.05 was considered statistically significant.

**Results:**

Twenty-nine cross-sectional studies with 13,957 study participants were included in this meta-analysis. The overall pooled prevalence of ITN utilization among pregnant women in Ethiopia was 51% (95% CI: 43–60). A statistically significant heterogeneity was observed across studies (*I*^2 ^= 99.09%; *P* < 0.001). Being literate [OR = 2.93 (95% CI: 2.14–4.01)], rural residence [OR = 1.76 (95% CI: 1.37–2.26)], and having knowledge of ITN [OR = 4.13 (95% CI: 1.57–10.81)] were factors significantly associated with ITN utilization among pregnant women.

**Conclusion:**

The utilization of ITNs among pregnant women was substantially lower than the national target, alarmingly highlighting the need for urgent and effective interventions. Maternal education status, place of residence, and knowledge of ITNs were independent predictors of ITN utilization. Health policymakers and programmers should design and implement the most effective strategies to scale up the utilization of ITNs by pregnant women and reduce malaria-related morbidity during pregnancy.

**Systematic Review Registration:**

CRD42022304432.

## Introduction

Malaria is a lethal disease that affects people all over the world, with tropical and sub-tropical regions having the highest prevalence (1). During pregnancy, the burden of malaria is doubled, with unfavorable maternal and neonatal outcomes (1). Pregnant women are more susceptible to malaria infections compared to non-pregnant women (2). Globally, around 228 million cases and 405,000 deaths from malaria were reported, with Africa accounting for 93% of cases and 94% of deaths (3). In sub-Saharan Africa, plasmodium falciparum, which causes 25% of all maternal deaths in malaria-endemic areas, is a major cause of maternal and newborn deaths (4, 5).

Malaria during pregnancy results in placental infection, leading to placental injury and insufficiency, which in turn causes low birth weight delivery and increases the risk of neonatal mortality (6). In addition, maternal anemia, intrauterine growth restriction, premature delivery, and stillbirth are adverse consequences of malaria infection (6, 7). Low birth weight (LBW) is an important predictor and etiology of infant mortality. Malaria in pregnancy is responsible for up to 900,000 LBW deliveries annually and over 100,000 infant deaths in Africa (8). Besides these, infants who survive LBW have higher morbidity, which increases the likelihood that they may experience delayed social and cognitive development (9, 10).

There have been increasing efforts to reduce the spread of malaria, particularly in sub-Saharan Africa. The key malaria prevention strategies in pregnancy are intermittent preventative treatment with anti-malarial medications and the regular and timely use of ITNs (11). Insecticide-treated bed net use is one of the most effective malaria-preventive methods during pregnancy (11, 12). If used properly and regularly, it decreases contact and infective mosquito bites by about 70%–90% (13). Studies have shown that ITN utilization in malaria-endemic areas can reduce malaria-related deaths by about 20% (14, 15).

The effective utilization of ITN remains a major global public health challenge, especially in developing nations like Ethiopia. Globally, the proportion of ITN utilization varies by region and assessment method, ranging from 6.8% to 82% (16–18). The proportion of ITN utilization in Ethiopia ranges from 28.1% to 81.6% (19, 20), with the lowest rates concentrated in the pastoralist community of Afar and the Somali region (21, 22). Several factors have been identified for the underutilization of ITNs, including lack of awareness of malaria prevention, inadequate numbers of nets available, lack of knowledge that ITN prevents malaria, inconvenience of using ITNs, unaffordability, and poor knowledge and attitude about ITN use (23–25).

Despite a few studies on ITN utilization among pregnant women in Ethiopia, there is a lack of cumulative evidence at the national level to show the exact extent and broader effects of ITN use. The lack of solid evidence necessitates continuing efforts to generate up-to-date information on ITN utilization that can be used in decision-making. Hence, this systematic review and meta-analysis were conducted to generate evidence-based, nationally reliable pooled evidence on ITN utilization and associated factors among pregnant women in Ethiopia.

This study provides nationally reliable pooled evidence on ITN utilization that could help health policymakers and program directors design effective strategies that focus on identified factors to increase the use of ITNs by pregnant women and reduce the morbidity and mortality associated with malaria during pregnancy.

## Methods

### The study protocol and registration

This systematic review and meta-analysis were conducted to determine the pooled prevalence of ITN utilization and associated factors among pregnant women in Ethiopia. This study was reported following the guidelines of the Preferred Reporting Items for Systematic Review and Meta-Analysis (PRISMA) checklists (26). All published articles that report on the prevalence of ITN utilization and associated factors among pregnant women were retrieved. The review was registered by the International Prospective Register of Systematic Reviews with the identification CRD42022304432.

### Eligibility criteria

This systematic review and meta-analysis included all primary cross-sectional studies conducted in Ethiopia. All original observational studies in Ethiopia that reported the prevalence of ITN utilization and associated factors among pregnant women were included. Both published and unpublished articles in English were retrieved and included in the review. However, studies that did not report the prevalence of ITN utilization and associated factors, studies that failed to report the primary outcomes of interest, case-control studies, case reports, qualitative studies, and articles without full texts were excluded.

### Outcome measures and definition of terms

This systematic review and meta-analysis had two main outcomes: The first outcome variable was to estimate the pooled prevalence of ITN utilization among pregnant women in Ethiopia, which was measured by the proportion of pregnant women who slept under ITNs during the night before the survey. The prevalence of ITN utilization among pregnant women reported in different studies was presented by pooling the prevalence of ITN utilization among pregnant women reported in the included articles. The second outcome was to identify the pooled effect of associated factors on ITN use among pregnant women in Ethiopia. Insecticide-treated nets refer to nets that have been treated with insecticide to kill mosquitoes and are used as physical barriers to prevent the bite of mosquitoes. Insecticide-treated net utilization is the use of properly hanged ITNs by pregnant women while sleeping.

### Search strategies

This systematic review and meta-analysis were conducted to estimate the pooled prevalence of insecticide-treated net utilization and associated factors among pregnant women in Ethiopia. All published articles reporting on insecticide-treated net utilization and associated factors among pregnant women in Ethiopia were retrieved from databases such as PubMed, CINAHL, Web of Science, Science Direct, SCOPUS, Google, and Google Scholar. All primary cross-sectional studies published before July 2022 were searched. The search was done using keywords such as insecticide-treated net, long-lasting insecticidal net, mosquito net, bed net, utilization, pregnant women, and Ethiopia. Combinations of Boolean operators (AND, OR), free keywords, and MeSH terms were used in the search process. For instance, PubMed search: (((“insecticidal” [All Fields] OR “insecticides” [All Fields] OR “insecticides” [Pharmacological Action] OR “insecticides” [MeSH Terms] AND “therapy” [MeSH Terms] OR “therapy” [All Fields] OR “therapeutics” [MeSH Terms] OR “therapeutics” [All Fields] OR “treating” [All Fields] OR “treated” [All Fields] AND “bed net” [All Fields] OR “long-lasting insecticidal net” [Title/Abstract] OR “mosquito net” [MeSH Terms] OR “insecticide-treated net” [All Fields] AND “pregnant women” [MeSH Terms] AND “Ethiopia” [MeSH Terms].

### Study selection and quality appraisal

All studies retrieved from all databases were exported to EndNote version X8, and duplicates were removed. Five independent reviewers (AD, IK, SH, KM, and AE) performed an initial review of articles by title and abstract to eliminate articles that were not obviously relevant to this review. Two reviewers (BE and BB) independently screened all articles for eligibility criteria. Any disagreements between the reviewers were solved through discussion or by the mediation of other reviewers (HB, AD, KM, SH, IK, and AE). Three reviewers (BB, BE, and IK) independently performed the full-text review. The quality of the articles was appraised independently by five reviewers (BB, BE, HB, IK, and AD) using the Newcastle-Ottawa quality assessment scale (27). The studies with a total score of 7 or higher on the quality evaluation checklist criteria were included in this systematic review and meta-analysis.

### Data extraction

Data were extracted from the full text of the retained articles by two reviewers (BB and BE) independently. A pre-defined Microsoft Excel 2010 format was used to extract the data from selected studies using the following headings: author's name, publication year, region, sampling methods, study design, sample size, response rate, and prevalence of ITN utilization. The accuracy of the data extraction was verified by comparing the results of the independently extracted data. The variations between the two reviewers were solved by re-extracting the data together, and the other reviewers checked the accuracy of the extracted data.

### Statistical analysis

The extracted data were exported to STATA Version 14.0 statistical software for synthesis and analysis. The random effect meta-analysis model was utilized to determine the study-speciﬁc true effects across studies. The forest plots were employed to show the pooled prevalence of ITN utilization and its associated factors among pregnant women in Ethiopia. The presence of heterogeneity was checked by the *I*^2^ test statistics at a *P*-value < 0.05 (28). The heterogeneity was performed and interpreted as low, moderate, and marked heterogeneity for *I*^2^ test values of 25, 50, and ≥75%, respectively. A subgroup analysis based on the study area (regions) and year of publications was performed to detect the source of heterogeneity. The potential publication bias was checked through visual inspection of the funnel plot and Egger's regression test (29). A random-effect meta-analysis model was used to show the pooled prevalence of ITN utilization among pregnant women. The size of the pooled effect of the factors associated with ITN utilization was estimated using an odds ratio with a 95% CI, and a *P*-value < 0.05 was considered statistically significant.

## Results

### Description of the studies

**Figure 1:**
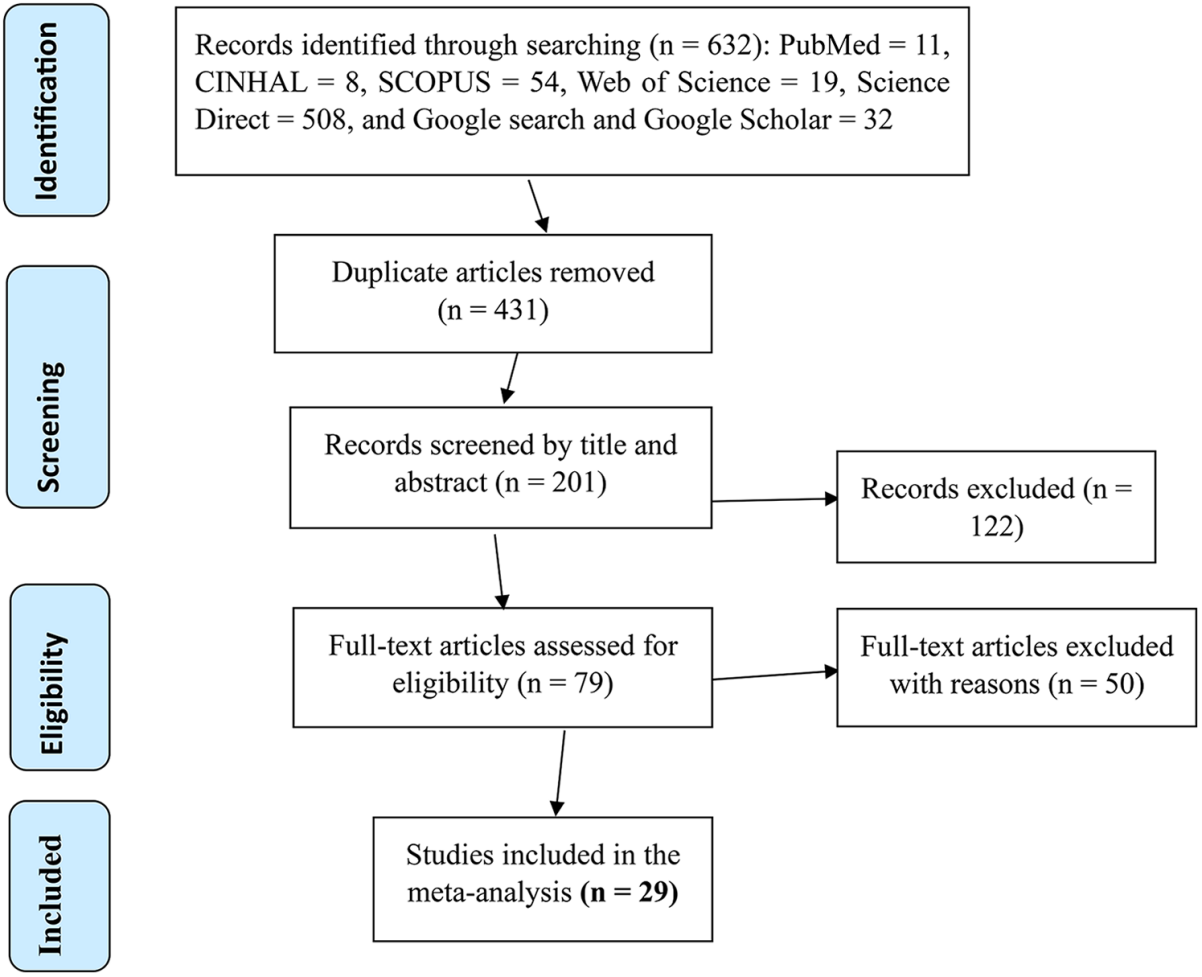
PRISMA flow diagram showing the selection process of eligible articles for the systematic review and meta-analysis of ITN utilization and associated factors among pregnant women in Ethiopia, 2022.

A total of 632 studies were identified from the database search. All studies were exported to the endnote library, and 431 duplicates were removed, while 122 studies were excluded after titles and abstracts were reviewed. Then, 79 studies were retrieved for detailed evaluation, and 50 studies were excluded after a full-text review due to different populations and failure to report outcomes of interest. Finally, 29 studies met the eligibility criteria and were included to estimate the pooled prevalence of ITN utilization among pregnant women in Ethiopia (Figure 1).

### Characteristics of the included articles

Twenty-nine (29) cross-sectional studies with a total of 13,957 study participants were included in this meta-analysis to determine the pooled prevalence of ITN utilization among pregnant women in Ethiopia. The prevalence of ITN utilization included in the review ranged from 18.9% to 88.3%. Of the 29 studies included in the final analysis, six were conducted in the Amhara region (30–35), seven in the Southern Nations, Nationalities, and Peoples' Region (SNNPR) (23, 36–41), eight in Oromia (24, 25, 35, 42–46), four in Tigray (19, 47–49), and the rest were in other regions in Ethiopia (20, 21, 50, 51). The minimum sample size was 20 participants in a study conducted in SNNPR (41) and the highest was 3,784 in a study conducted in three districts of the Jimma zone, Oromia region (43) (Table 1).

**Table 1:** General characteristics of studies included in the systematic review and meta-analysis among pregnant women in Ethiopia, 2022.

Authors	Publication year	Region	Sample size	Event %	Study design	Sampling methods	Study subjects
Yirsaw et al. (30)	2021	Amhara	724	56.50	CS	Simple random	Pregnant mothers
Yitayew et al. (32)	2018	Amhara	226	70.80	CS	Systematic	Pregnant mothers
Gultie et al. (36)	2020	SNNPR	2657	47.20	CS	Simple random	Pregnant mothers
Angesom et al. (19)	2020	Tigray	550	63.10	CS	Systematic	Pregnant mothers
Watiro and Awoke (20)	2016	Gambela	690	51.40	CS	Simple random	Pregnant mothers
Yeshaneh and Adane (37)	2020	SNNPR	93	70.80	CS	Simple random	Pregnant mothers
Shonga et al. (38)	2018	SNNPR	630	72.50	CS	Systematic	Pregnant mothers
Belay and Deressa (47)	2008	Tigray	481	58.40	CS	Simple random	Pregnant mothers
Felema (25)	2007	Oromia	116	37.10	CS	Simple random	Pregnant mothers
Nesga et al. (42)	2020	Oromia	110	61.00	CS	Simple random	Pregnant mothers
Tadele et al. (44)	2014	Oromia	65	43.10	CS	Systematic	Pregnant mothers
Araya et al. (49)	2015	Oromia	120	69.00	CS	Systematic	Pregnant mothers
Abitew et al. (34)	2020	Amhara	626	88.30	CS	Systematic	Pregnant mothers
Tariku et al. (31)	2020	Amhara	417	33.60	CS	Systematic	Pregnant mothers
Fuge et al. (23)	2015	SNNPR	422	21.40	CS	Simple random	Pregnant mothers
Legesse et al. (51)	2008	Benishangul	66	25.70	CS	Systematic	Pregnant mothers
Tesfaye et al. (46)	2022	Oromia	424	39.90	CS	Systematic	Pregnant mothers
Alemu et al. (33)	2018	Amhara	260	58.80	CS	Systematic	Pregnant mothers
Gontie et al. (50)	2020	Benishangul	447	71.30	CS	Simple random	Pregnant mothers
Negash et al. (21)	2012	Afar	210	79.00	CS	Systematic	Pregnant mothers
Dagne and Deressa (39)	2008	SNNPR	63	74.60	CS	Systematic	Pregnant mothers
Hambisa et al. (45)	2018	Oromia	177	52.50	CS	Systematic	Pregnant mothers
Gobena et al. (24)	2012	Oromia	145	37.20	CS	Simple random	Pregnant mothers
Deressa et al. (40)	2014	SNNPR	83	39.80	CS	Simple random	Pregnant mothers
Deressa et al. (35)	2011	Oromia	203	18.90	CS	Simple random	Pregnant mothers
Deressa et al. (35)	2011	Amhara	66	36.40	CS	Simple random	Pregnant mothers
Astatkie and Feleke (41)	2009	SNNPR	20	35.00	CS	Simple random	Pregnant mothers
Berhane and Ahmed	2008	Tigray	82	57.30	CS	Systematic	Pregnant mothers
Ouedraogo et al. (43)	2019	Oromia	3,784	26.30	CS	Simple random	Pregnant mothers

SNNPR, southern nations, nationalities, and peoples’ region; CS, cross-sectional.

### Pooled prevalence of ITN utilization

**Figure 2:**
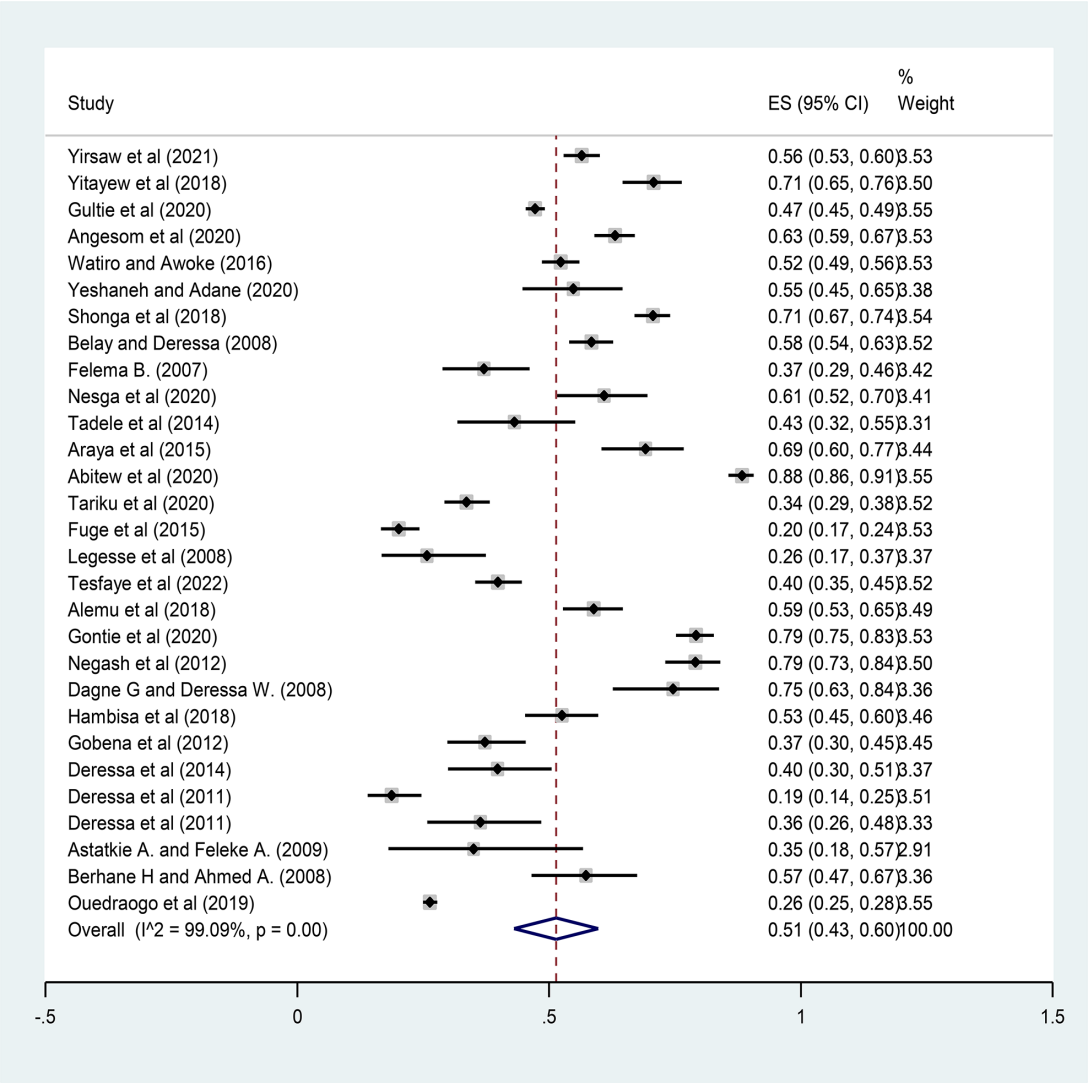
Forest plot for the pooled prevalence of ITN utilization among pregnant women in Ethiopia, 2022.

The overall pooled prevalence of ITN utilization among pregnant women in Ethiopia was 51% (95% CI: 43–60). A statistically significant heterogeneity was observed across studies (*I*^2 ^= 99.09%; *P* < 0.001) (Figure 2).

### Publication bias

**Figure 3:**
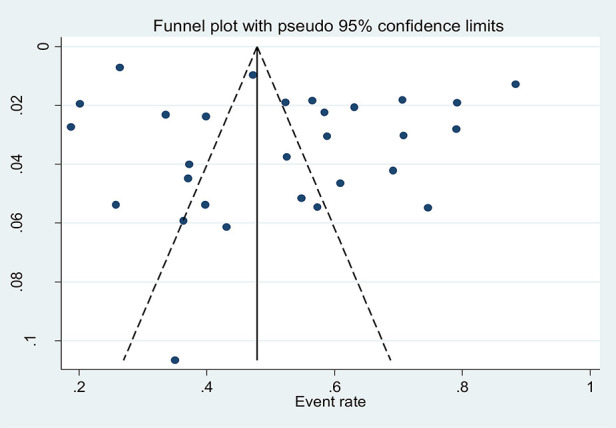
Funnel plot showing the symmetric distribution of articles on the pooled prevalence of ITN utilization among pregnant women in Ethiopia, 2022.

The asymmetrical distribution of the funnel plot was visually inspected to see the presence of publication bias among studies and indicated no significant publication bias due to the small sample size effect (Figure 3). Additionally, Egger's regression test was performed, showing no evidence of significant publication bias among studies (*P* = 0.230).

### Subgroup analysis

**Figure 4:**
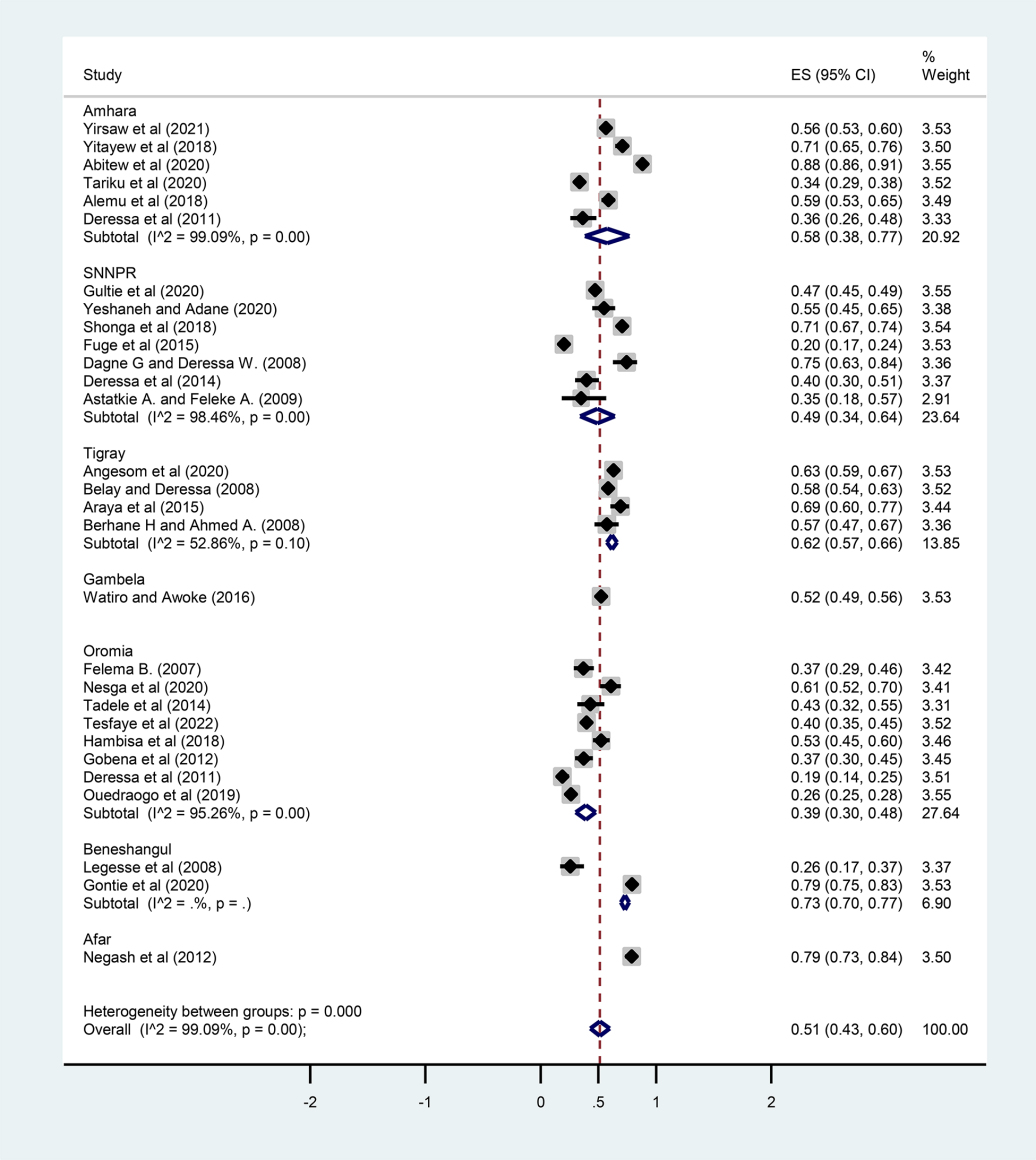
Subgroup analysis of the prevalence of ITN utilization by regions (study area) among pregnant women in Ethiopia, 2022.

**Figure 5:**
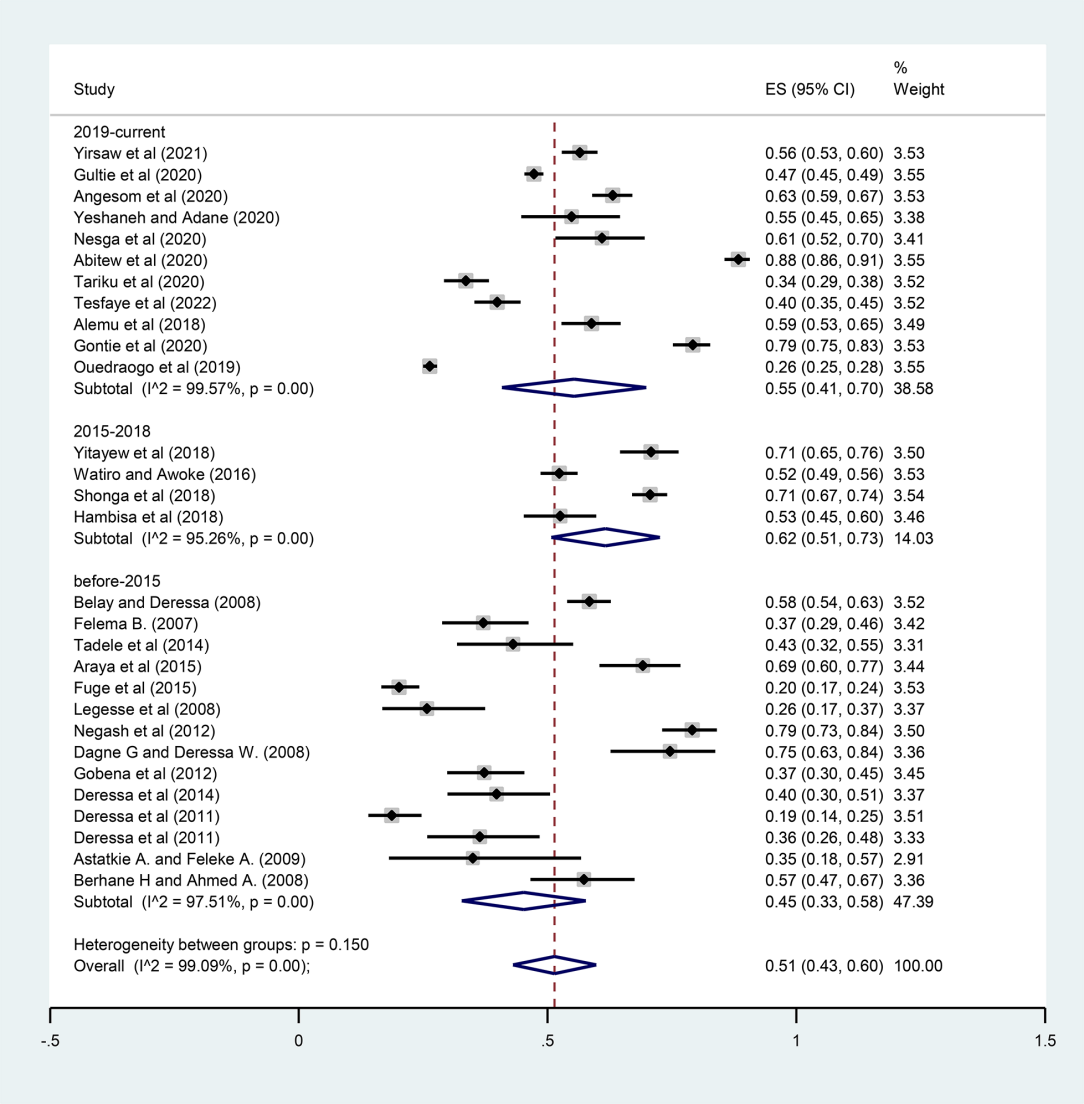
Subgroup analysis of the prevalence of ITN utilization by year of publications among pregnant women in Ethiopia, 2022.

A subgroup analysis was performed based on the study area (regions) and publication year to identify the possible source of heterogeneity. A statistically significant heterogeneity was observed between study groups (*I*^2 ^= 99.09%; *P* < 0.001). In this pooled analysis, the Tigray region had the highest prevalence of ITN utilization (62%; 95% CI: 0.57–0.66) while Oromia had the lowest prevalence (39%; 95% CI: 0.30–0.48) (Figure 4). Regarding the publication year, the prevalence of ITN utilization among pregnant women before 2015 was 45% (95% CI: 0.33–0.58), increased to 62% (95% CI: 0.51–0.73) between 2015 and 2018, and decreased to 55% (95% CI: 0.41–0.70) between 2019 and 2021 (Figure 5).

### Factors associated with ITN utilization

Maternal age, educational status, place of residence, monthly income, knowledge of ITNs, and distance from a health facility were extracted in this review to identify factors associated with ITN use. Variables that had statistically significant associations with ITN utilization in at least three studies were included in this meta-analysis. Thus, maternal educational status, place of residence, and knowledge of ITNs were identified as independent predictors of ITN utilization.

#### Educational status and ITN utilization

**Figure 6:**
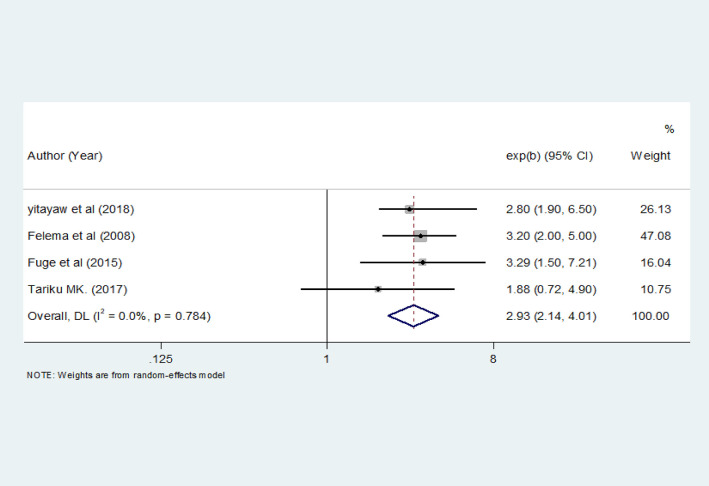
The pooled odds ratio of the association between education status and ITN utilization among pregnant women in Ethiopia, 2022.

The education status of mothers was significantly associated with ITN utilization in four studies (23, 25, 31, 32). A total of 1,181 pregnant women were included to determine the association between education status and ITN utilization. The pooled odds ratio showed that literate pregnant women were 2.93 times more likely to utilize ITN than illiterate pregnant women [OR = 2.93 (95% CI: 2.14–4.01)] (Figure 6).

#### Place of residence and ITN utilization

**Figure 7:**
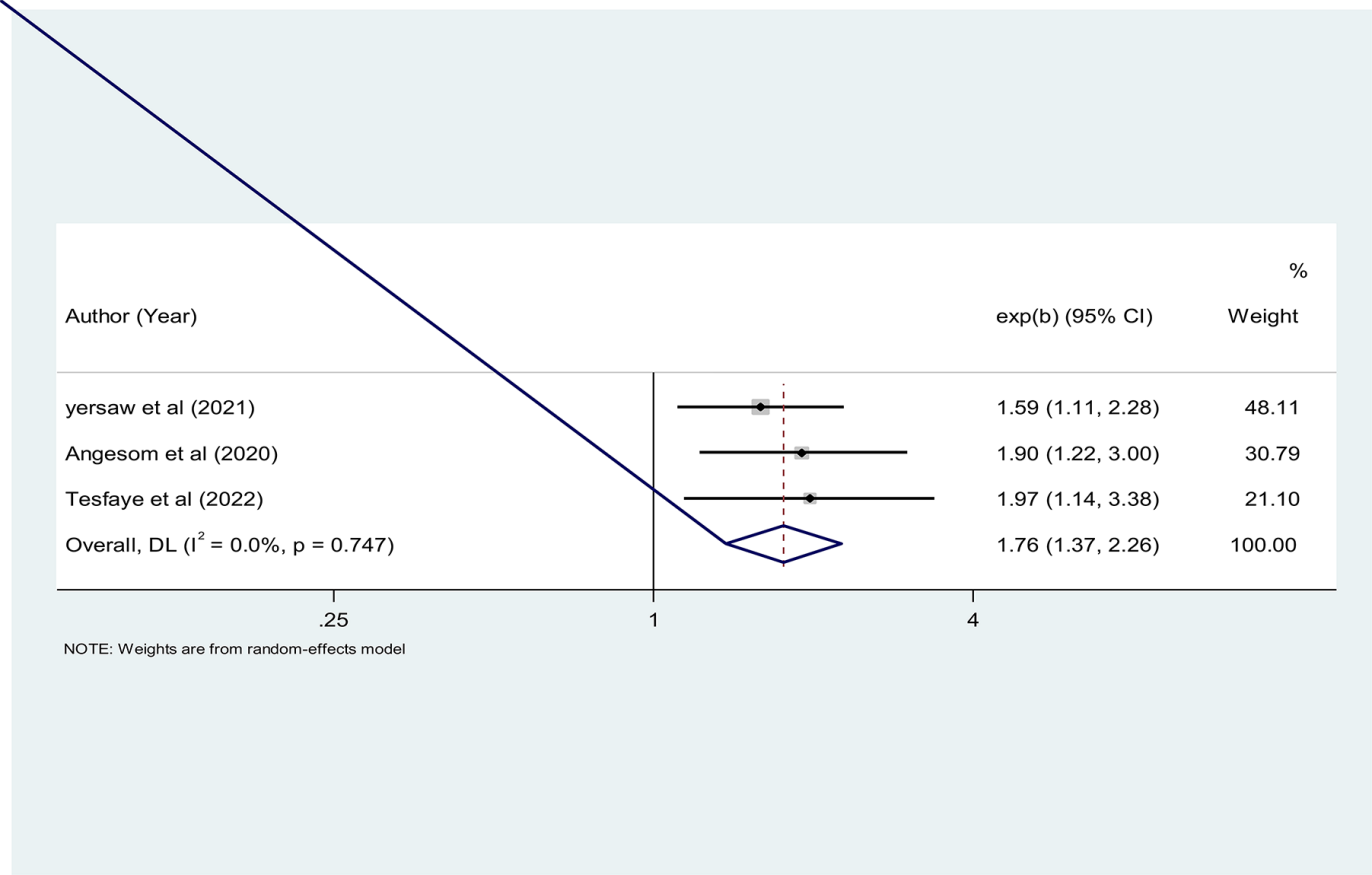
The pooled odds ratio of the association between place of residence and ITN utilization among pregnant women in Ethiopia, 2022.

The place of residence was significantly associated with ITN utilization in three studies (19, 30, 46). A total of 2,513 pregnant women were included to analyze the association between place of residence and ITN utilization. The pooled result showed that pregnant women who live in rural areas were 1.76 times more likely to utilize ITN than pregnant women living in urban areas [OR = 1.76 (95% CI: 1.37–2.26)] (Figure 7).

#### Knowledge of mothers and ITN utilization

**Figure 8:**
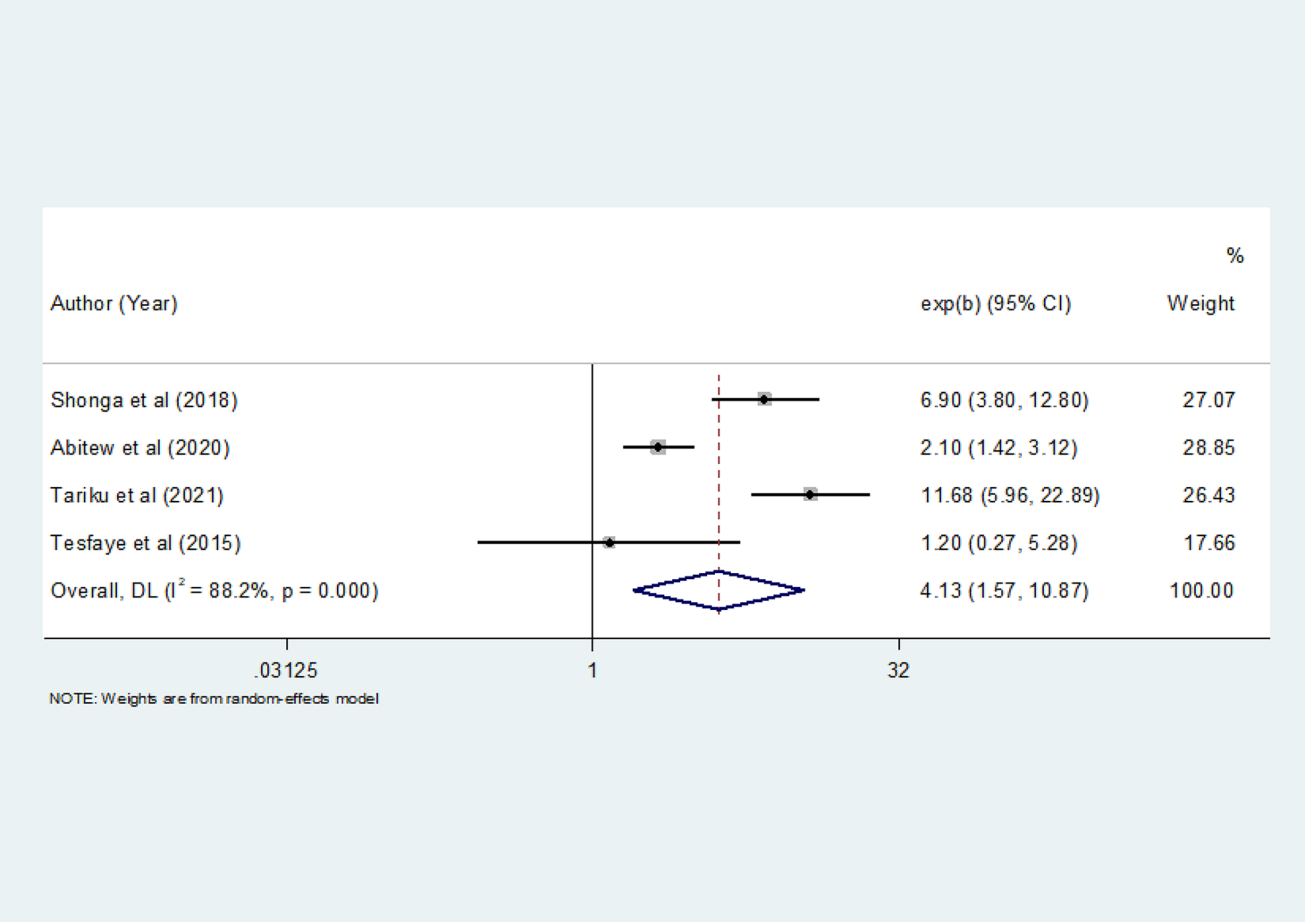
The pooled odds ratio of the association between level of knowledge and ITN utilization among pregnant women in Ethiopia, 2022.

Maternal knowledge of ITNs had a significant statistical association with ITN utilization in four studies (31, 34, 35, 38). A total of 1,697 pregnant women were included to show the association between knowledge and ITN utilization. The pooled odds ratio showed that pregnant mothers who had knowledge of ITNs were 4.13 times more likely to utilize ITN than pregnant mothers who had no knowledge [OR = 4.13 (95% CI: 1.57–10.81)] (Figure 8).

## Discussion

This systematic review and meta-analysis were conducted to determine the pooled prevalence of ITN utilization and its associated factors among pregnant women in Ethiopia. The pooled prevalence of ITN utilization among pregnant women in the country was very low. The result is significant and calls for an urgent national-level appropriate strategy for the prevention of further malaria-related complications, for both pregnant women and newborns, and to reduce morbidity and mortalities of both groups caused by malaria infection.

The findings from this systematic review and meta-analysis showed that the overall pooled estimate of ITN utilization among pregnant women in Ethiopia was 51% (95% CI: 43–60). This implies that 51 out of 100 pregnant women slept under ITNs before the study to prevent mosquito bites. The result of this meta-analysis is consistent with the findings from the 2015 national malaria indicators survey in Ethiopia (44%) (52) and a review in sub-Saharan Africa (58.3%) (53). However, this is low compared to the reviews in sub-Saharan Africa: Malawi (81%), Zambia (80%), and Senegal (78%) (54), and is higher than the findings from Uganda (35%) (55) and Nigeria (43.3%) (56). This could be attributed to the variability in the number of studies included in the review and the differences in sociodemographic characteristics, beliefs, and other cultural differences among participants across the countries.

The utilization of ITN in this meta-analysis revealed considerable regional variations in Ethiopia. In this pooled analysis, 62% of pregnant women slept under ITNs in the Tigray region, 58% in Amhara, 49% in SNNPR, and 39% in Oromia. The highest proportion of ITN utilizers were from the Tigray region (62%), and the lowest proportion were from Oromia (39%). This variation might be due to differences in weather conditions, environmental factors, malaria preventive strategies, and mosquito population densities. Thus, ITN use is thought to be higher in areas with high mosquito populations compared to areas with lower mosquito populations (21, 22).

This systematic review and meta-analysis identified maternal knowledge of ITNs, education status, and place of residence as factors significantly associated with ITN utilization. In this meta-analysis, pregnant mothers who had knowledge of ITNs were 4.13 times more likely to utilize ITN than pregnant mothers who had no knowledge. This finding is consistent with studies conducted on the utilization of ITN during pregnancy in Ethiopia (31, 35, 38). This finding indicates the need for effective and evidence-based interventions to boost ITN utilization and reduce malaria-related morbidity. Additionally, behavioral change interventions should be made available to encourage pregnant women to utilize ITN.

The findings of this meta-analysis also revealed that maternal education status had an association with ITN utilization. The pooled odds ratio showed that literate pregnant women were 2.93 times more likely to utilize ITN than illiterate pregnant women. This result is in line with studies conducted on ITN utilization during pregnancy in Ethiopia (25, 31, 32). This is because mothers with a high educational status know more about the use and importance of ITNs to prevent malaria. This suggests that the level of education has an impact on the utilization of malaria prevention and control interventions.

Furthermore, the findings of this meta-analysis showed that pregnant women who live in rural areas were 1.76 times more likely to utilize ITN than pregnant women living in urban areas. This finding is consistent with studies conducted in Ethiopia (30, 46). This might be due to the fact that most rural villages in Ethiopia are found around water sources such as rivers or someplace where the government has built dams in rain-deficient areas. Consequently, these stagnant water sources could create a favorable condition for mosquito breeding, which would force people to utilize ITN.

In this meta-analysis, significant heterogeneity was observed as a result of the variations between the studies. To manage it, we performed a random effect model analysis. Subgroup analysis by study area (regions) and publication year was also performed to find the source of heterogeneity. The heterogeneity in estimated prevalence could be related to variations in the study period and area. The study sample differences may additionally contribute to the heterogeneity.

### Strengths and limitations

The investigators used extensive and comprehensive search strategies in multiple databases. Studies were evaluated for methodological quality using a standardized tool. On the other hand, the study had some limitations: All studies included in this systematic review and meta-analysis used cross-sectional study designs, making it difficult to establish cause-and-effect relationships between variables. The presence of significant heterogeneity among studies was another limitation. Another limitation is that studies published in languages other than English were not included in this meta-analysis and are also considered a limitation of this study.

## Conclusion

This systematic review and meta-analysis showed that a substantial proportion of pregnant women did not sleep under ITN during pregnancy, alarmingly highlighting the need for urgent and effective interventions. Maternal educational status, place of residence, and knowledge of ITNs were independent predictors of ITN utilization among pregnant women. Understanding the determinants of ITN utilization will aid in designing and implementing policies that can reduce and alleviate malaria infections and related complications. Therefore, health policymakers and programmers working on malaria prevention should design and implement appropriate strategies to scale up the utilization of ITNs by pregnant women and reduce malaria-related morbidity and mortality. Community health education should also be provided, especially for vulnerable groups, to increase the use of ITNs during pregnancy.

## Data Availability

The original contributions presented in the study are included in the article/Supplementary Material, further inquiries can be directed to the corresponding author.
